# Editorial: Innovation and transformation of chronic airway diseases

**DOI:** 10.3389/fmolb.2022.1089813

**Published:** 2022-12-13

**Authors:** Rui Zhao, Yan Chen, Yiming Ma, Chunbin Zou, Jing Zhang, Zhihua Chen

**Affiliations:** ^1^ Second Xiangya Hospital, Central South University, Changsha, China; ^2^ Division of Pulmonary, Allergy, and Critical Care Medicine, Department of Medicine, University of Pittsburgh School of Medicine, Pittsburgh, PA, United States; ^3^ Zhongshan Hospital, Fudan University, Shanghai, China; ^4^ Second Affiliated Hospital, School of Medicine, Zhejiang University, Hangzhou, China

**Keywords:** chronic airway disease, innovation, sulfur compounds, screening, advances

Chronic airway disease, including chronic obstructive pulmonary disease (COPD), bronchial asthma, bronchiectasis, and other pulmonary disease, is a common respiratory disease worldwide (Soriano et al., 2020, 8(6), 585–596).COPD was the third leading cause of death worldwide, causing the death of 3.23 million people (WHO, 2019), which has become a huge global burden (Quaderi and Hurst, 2018, 3, e4). In this context, numerous efforts have been made with the aim of identifying the mechanism of these diseases, exploring effective prevention measures and therapeutic strategies. In particular, innovation and transformation of chronic airway diseases should be worthy of attention. To date, the mechanism of chronic airway disease is not completely understood (Barnes Peter, 2017). Nonetheless, diverse researches have shown great advances, which may bring more efficient therapy of these diseases. Despite this, more innovation is necessary for us to solve the problems in treatment and prevention of chronic airway diseases. This Research Topic aims to collect innovation and transformation research in fields related to the pathogenesis and treatment of chronic airway diseases, to provide new alternatives for early diagnosis and treatment of chronic airway diseases. In this Research Topic, Qiao et al. reviewed the regulator roles of the lncRNA – miRNA – mRNA network in different cell types and their potential as the biomarkers or therapeutic targets of chronic inflammatory airway diseases (Qiao et al.). Several lncRNAs acting as miRNA sponges in different cells, such as bronchial epithelial cells, pulmonary microvascular endothelial cells and T Lymphocytes, have been focused in chronic airway diseases. The cross-talk among lncRNAs, miRNAs and mRNAs is involved in the pathophysiology of chronic inflammatory airway diseases. Meanwhile, Jiang and Chen summarized and analyzed the biological functions and mechanisms of sulfur compounds in regulating COPD and its comorbidities (Jiang and Chen). Sulfur compounds have the potential to protect individuals from developing chronic inflammatory diseases due to their antioxidant and anti-inflammation capabilities. The effect of these sulfur compounds on the development and treatment of COPD discussed in this review. Similarly, Lin et al. assessed endoplasmic reticulum (ER) stress markers, Epithelial mesenchymal transition (EMT) markers and associated signal molecules in rat lungs, bronchial epithelial cells, and human peripheral lung tissues to investigate the effect of H_2_S in regulating EMT and the underlying mechanisms (Lin et al.). They found that H_2_S inhibits cigarette smoke (CS)- or nicotine- induced ER stress and EMT in bronchial epithelial cells and alleviates CS-induced lung tissue damage and small airway fibrosis. The research suggested endogenous H_2_S may have an inhibitory effect on EMT related small airway fibrosis through regulating ER stress. The inositol-requiring enzyme 1 (IRE1) signal pathway and its downstream signal molecule p-Smad2/3 may be responsible for the inhibitory effect of H_2_S. On the other hand, Huang et al. performed untargeted metabolomics analysis of lung tissue and plasma sample, characterized the metabolic profile of lung tissue from COPD patients and found that glycerophospholipids (GPs) and amino acids metabolism disorder in male COPD patients. GPs may be related to COPD by participating in oxidative stress and inflammation (Huang et al.). Huang et al. screened and validated differential metabolites associated with male COPD and disordered metabolic pathways. They screened two two overlapping metabolites, phytosphingosine and L-tryptophan, as new promising biomarkers, could be two indicators for early screening of COPD patients. Zhang et al. investigated the effect of glycyl -L histidyl - L-lysine-Cu2+(GHK-Cu) on emphysema induced by CS (Zhang et al.). GHK-Cu treatment attenuated the CS-induced emphysematous changes and partially reversed the matrix metalloprotein -9 (MMP-9)/tissue inhibitor of metalloproteinases-1 (TIMP-1) imbalance in the lung tissue. They found that GHK-Cu provides potential protection against oxidative stress and inflammation induced by CS exposure the protective effects are likely related to modulation of the NF-κB and Nrf2/Keap1 pathways. This report is the first to stress the role of GHK-Cu in an animal model of CS-induced emphysema. GHK-Cu might be a therapeutic candidate for COPD with the role of anti-inflammation and anti-oxidation.

In summary, lots of studies in this Research Topic discuss the mechanism of chronic airway diseases and demonstrate innovative targets and biomarkers. The Research Topic reports the regulator roles of different biomolecules in chronic airway diseases, provides potential treatment and prevention ideas. The innovation and transformation of chronic airway diseases deserve more attention.
